# Supplementary material for: Differential Effects of Pancreatic Cancer-Derived Extracellular Vesicles Driving a Suppressive Environment
Source: Int J Mol Sci. 2023 Sep 27;24(19):14652. doi: 10.3390/ijms241914652 (PMC10572854; doi:10.3390/ijms241914652)
Supplement: Supplementary file 1 [file ijms-24-14652-s001.zip › ijms-2614873-supplementary.pdf]

Supplementary Figure S1. Graphical Abstract: Immunosuppressive role of HA-PDAC sEVs in the tumor microenvironment.

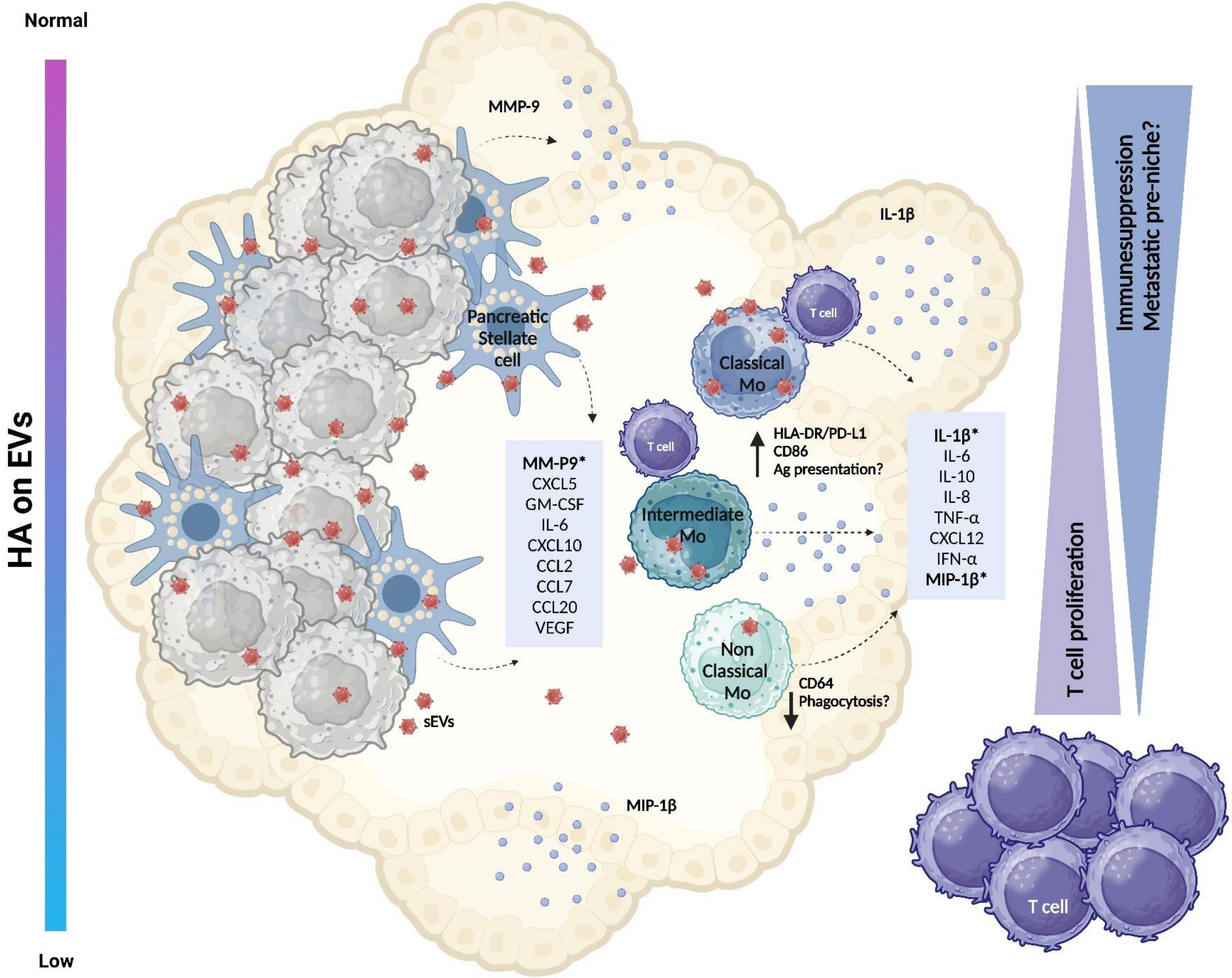

Supplemental Figure S2

A.

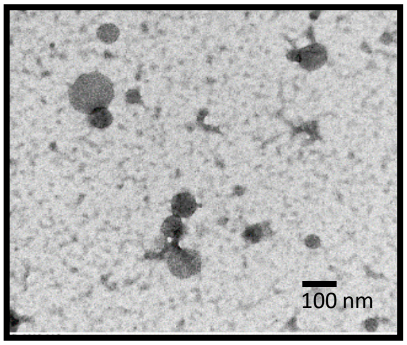

B.

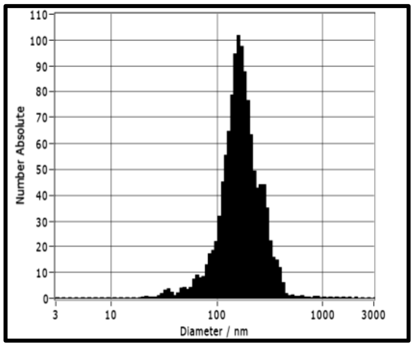

C.

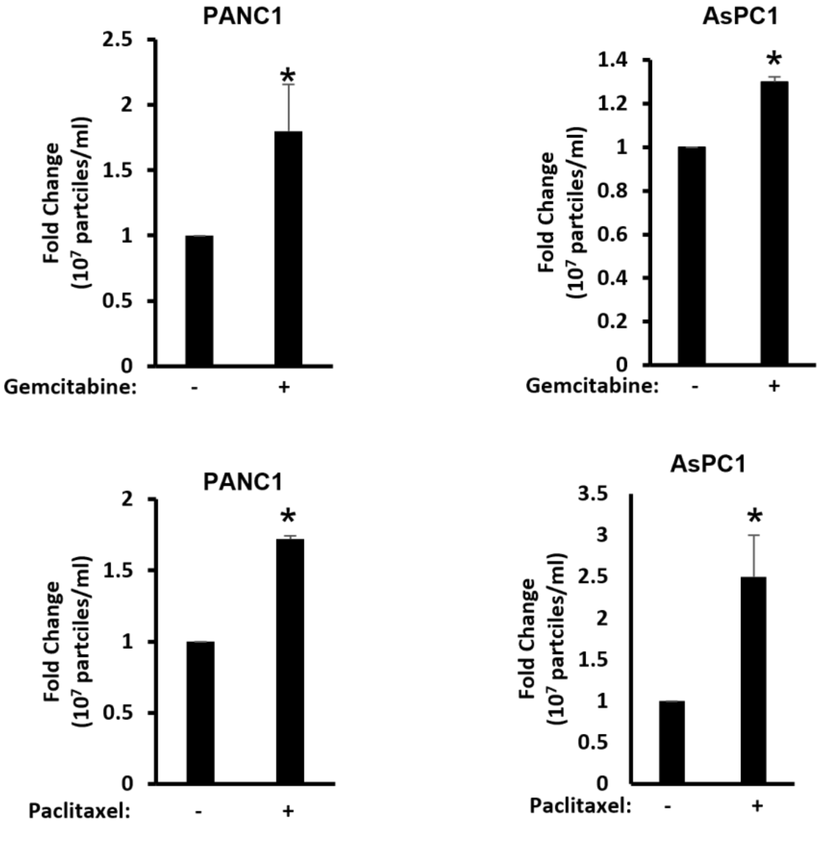

Supplemental Figure S3

A.

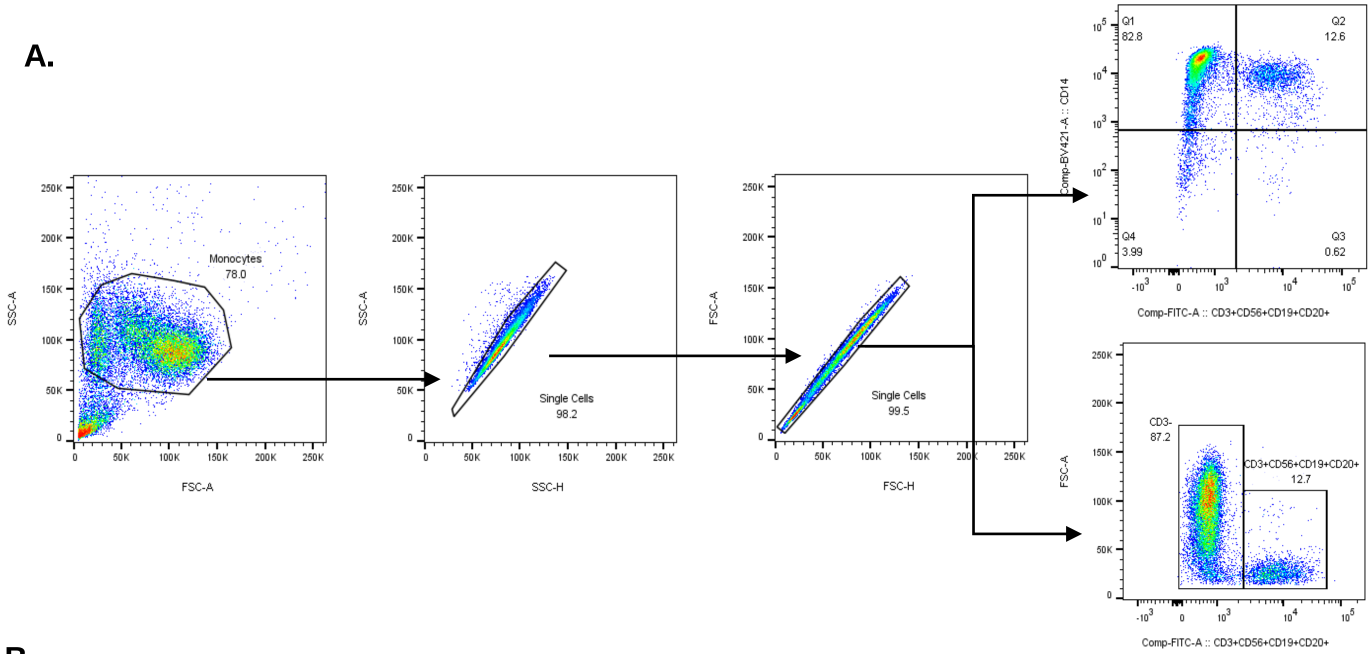

B.

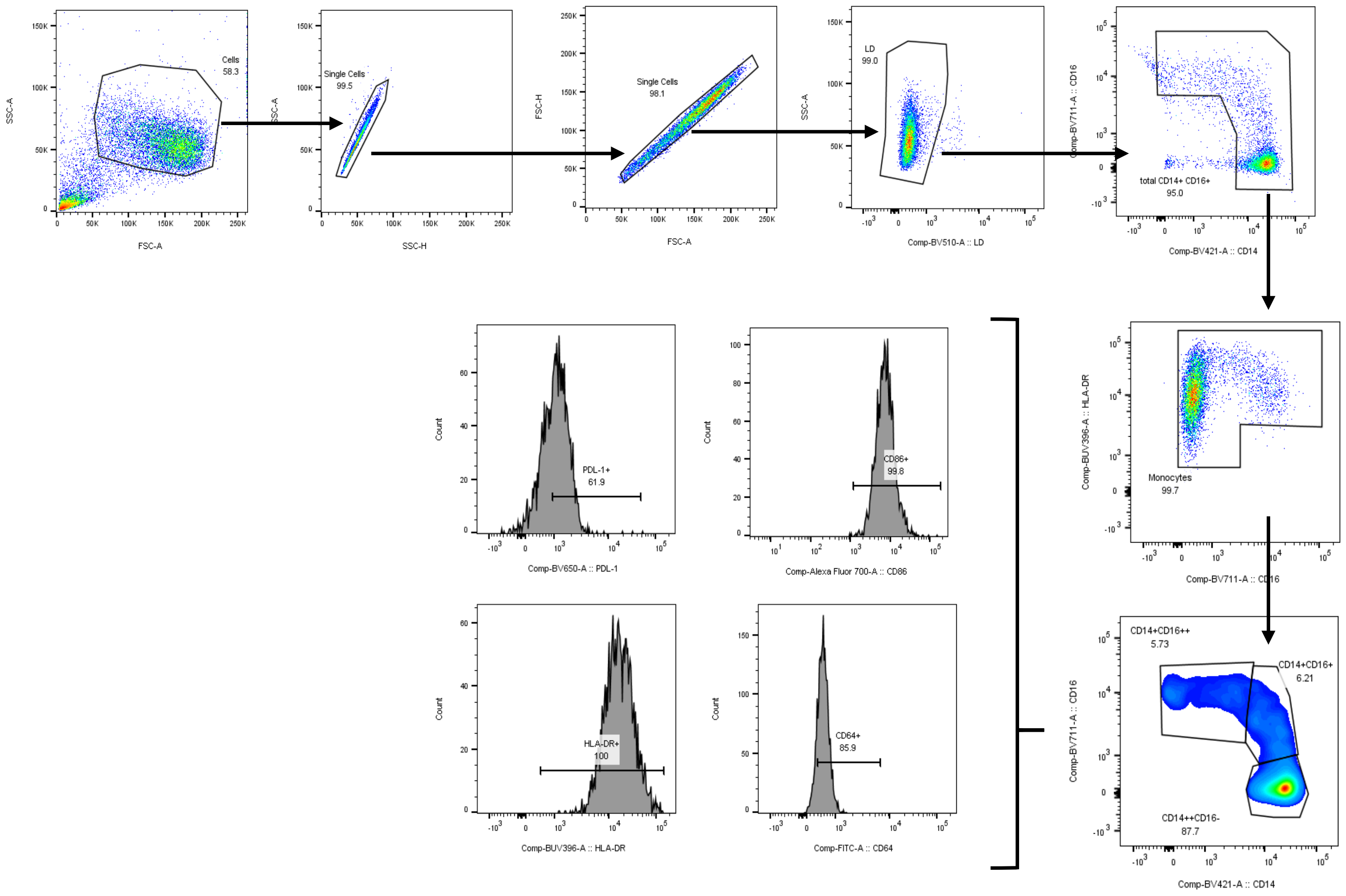

Supplemental Figure S4

A.

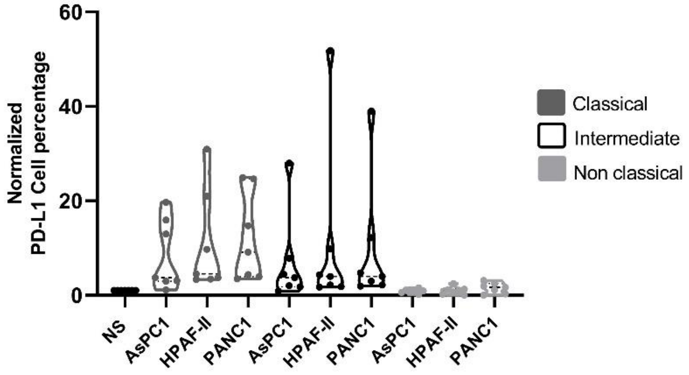

B.

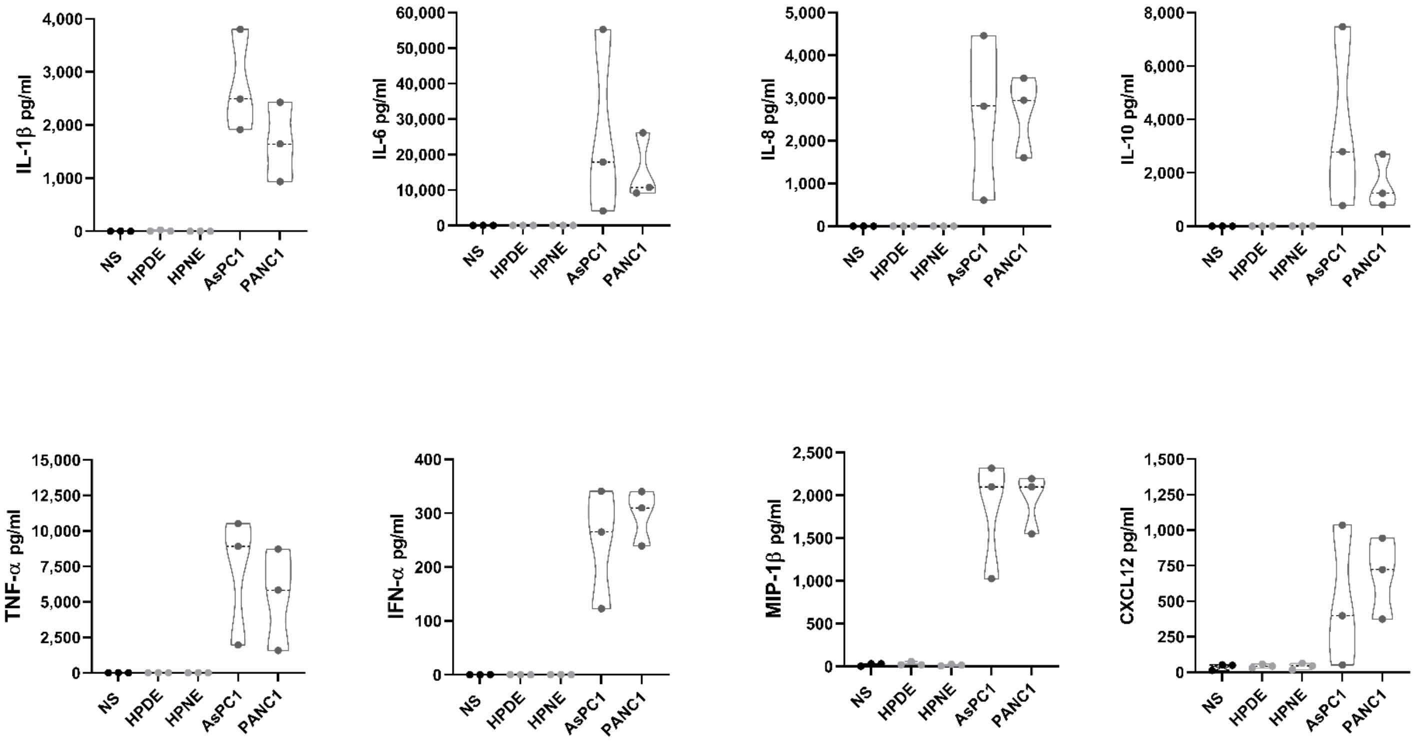

Supplemental Figure S5

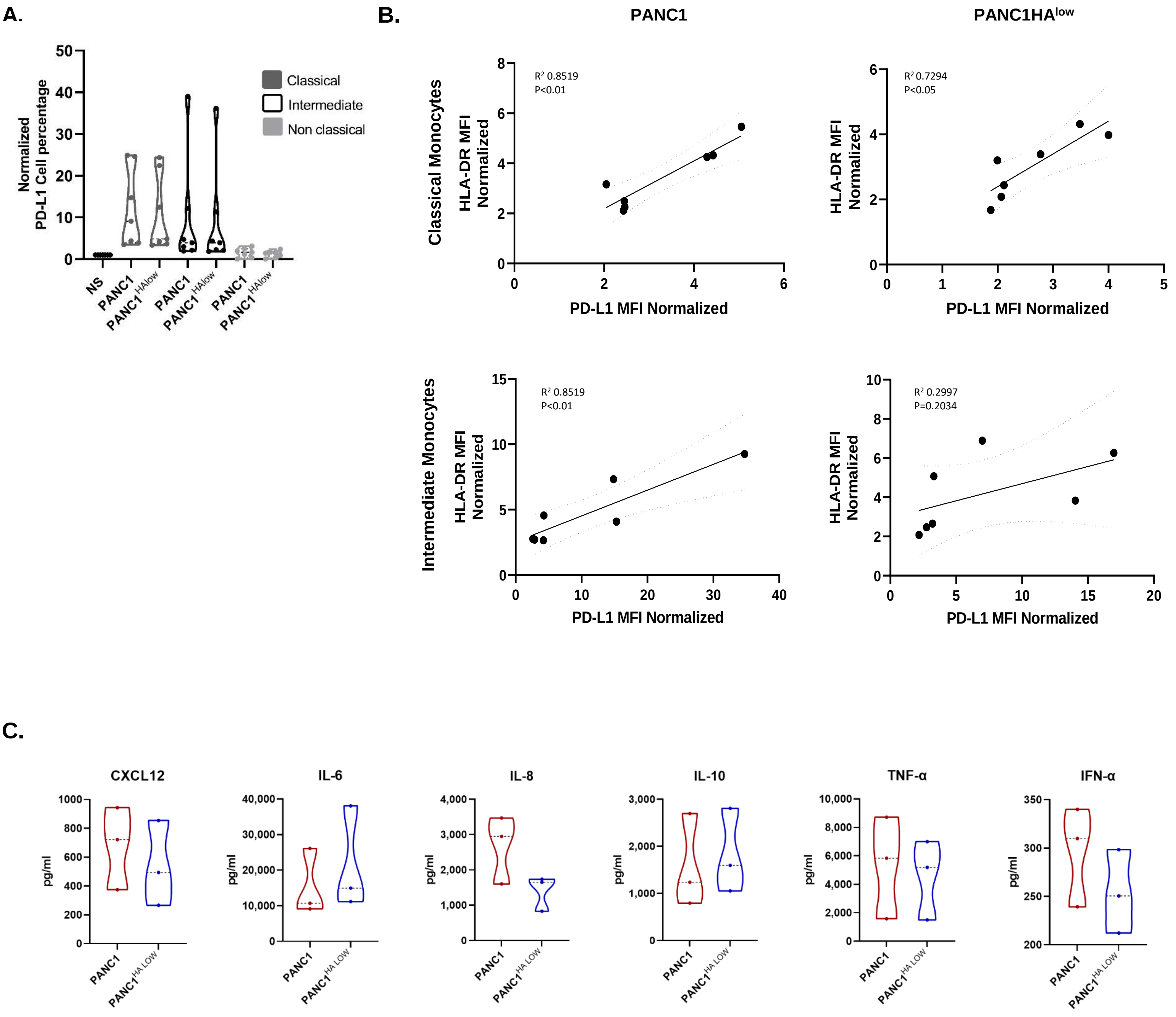

Supplemental Figure S6.

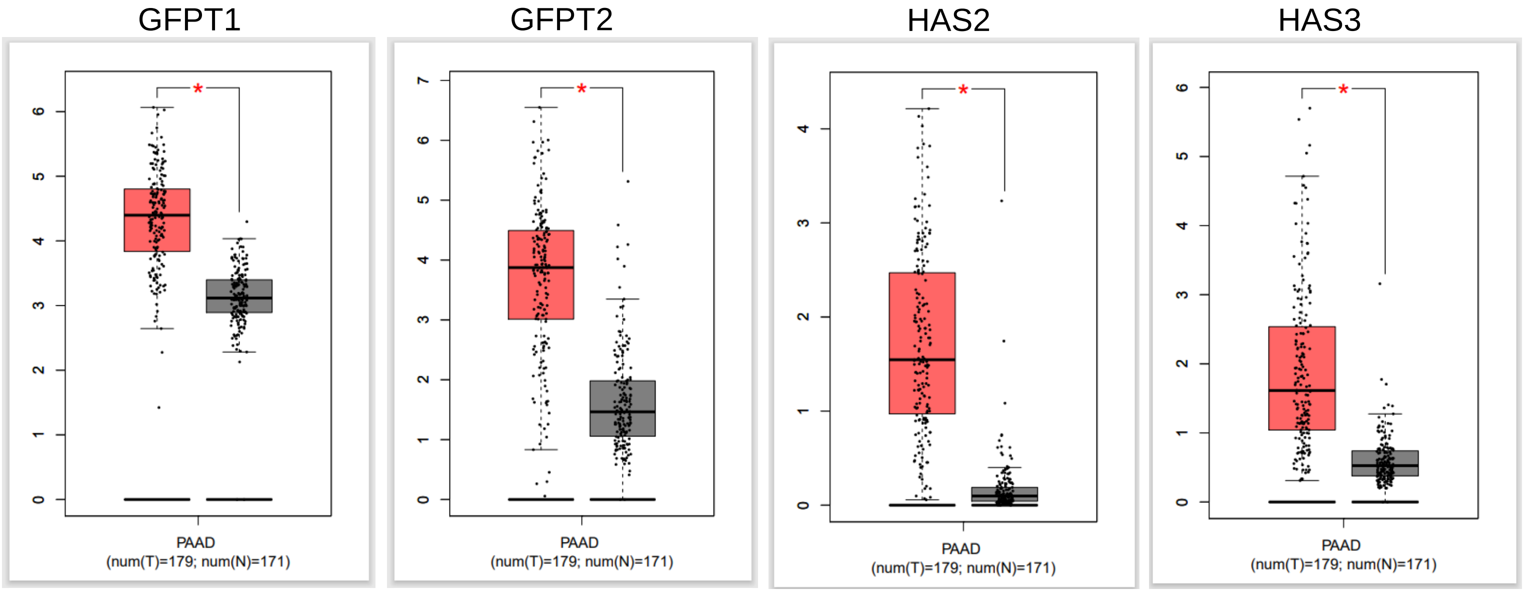

Supplementary Table S1. Multiple comparison statistics from Cytokine quantification by Luminex

	IL-1β		IL-6		IL-8		IL-10		TNF-α		IFN-α		MIP-1α		CXCL12	
Tukey's multiple comparisons test	Adjusted P Value		Adjusted P Value		Adjusted P Value		Adjusted P Value		Adjusted P Value		Adjusted P Value		Adjusted P Value		Adjusted P Value	
NS vs. HPDE	ns	>0.9999	ns	>0.9999	ns	>0.9999	ns	>0.9999	ns	>0.9999	ns	>0.9999	ns	>0.9999	ns	>0.9999
NS vs. HPNE	ns	>0.9999	ns	>0.9999	ns	>0.9999	ns	>0.9999	ns	>0.9999	ns	>0.9999	ns	>0.9999	ns	>0.9999
NS vs. PANC-1	*	0.017	ns	0.6963	*	0.0324	ns	0.7884	ns	0.197	***	0.0002	**	0.0011	ns	0.0954
NS vs. ASPC1	***	0.0003	ns	0.2159	*	0.0359	ns	0.091	ns	0.053	**	0.0012	**	0.0021	ns	0.3418
NS vs. PANC$^{HA\ LOW}$	ns	0.6113	ns	0.3799	ns	0.4463	ns	0.6814	ns	0.3366	***	0.0008	****	<0.0001	ns	0.261
HPDE vs. HPNE	ns	>0.9999	ns	>0.9999	ns	>0.9999	ns	>0.9999	ns	>0.9999	ns	>0.9999	ns	>0.9999	ns	>0.9999
HPDE vs. PANC-1	*	0.0175	ns	0.6967	*	0.0324	ns	0.7884	ns	0.1979	***	0.0002	**	0.0012	ns	0.0999
HPDE vs. ASPC1	***	0.0003	ns	0.2161	*	0.0359	ns	0.091	ns	0.0533	**	0.0012	**	0.0022	ns	0.3547
HPDE vs. PANC$^{HA\ LOW}$	ns	0.6224	ns	0.3802	ns	0.4463	ns	0.6814	ns	0.3379	***	0.0008	****	<0.0001	ns	0.2716
HPNE vs. PANC-1	*	0.0169	ns	0.6963	*	0.0324	ns	0.7884	ns	0.1972	***	0.0002	**	0.0011	ns	0.0982
HPNE vs. ASPC1	***	0.0003	ns	0.2159	*	0.0359	ns	0.091	ns	0.0531	**	0.0012	**	0.002	ns	0.3499
HPNE vs. PANC$^{HA\ LOW}$	ns	0.6088	ns	0.3799	ns	0.4463	ns	0.6814	ns	0.3368	***	0.0008	****	<0.0001	ns	0.2676
PANC-1 vs. ASPC1	ns	0.1809	ns	0.9131	ns	>0.9999	ns	0.5485	ns	0.9583	ns	0.8109	ns	0.9984	ns	0.9507
PANC-1 vs. PANC$^{HA\ LOW}$	ns	0.2316	ns	0.9908	ns	0.5471	ns	>0.9999	ns	0.9987	ns	0.9128	*	0.034	ns	0.9837
ASPC1 vs. PANC$^{HA\ LOW}$	**	0.0035	ns	0.9981	ns	0.5811	ns	0.662	ns	0.8298	ns	0.9998	*	0.0175	ns	>0.9999

Data From 3 Normal donors, results from triplicate lysates and supernatant after overnight incubation of monocytes without or with EVs. One-way ANOVA test, post hoc Tukey's multiple comparison test, alpha threshold 0.05 (95% confidence interval). * $p < 0.05$, ** $p < 0.01$, *** $p < 0.001$, **** $p < 0.0001$.

Supplemental Table S2: Clinical Summary of PDAC Patients

**Patients**	**Pathological stage***	**Diabetes Mellitus**	**Metastasis**	**Post treatment timepoint**	**Treatment**	**HA (ng/100µg protein)**	**Glucose (mg/dl)**
1A	II B	Yes	-	Post-op	First line neoadjuvant FOLFIRINOX, followed by capectiabine XRT 50.4Gy/28 fxs	**4**	92
1B						**5.9**	115
2A	II B	No	-	Post-op	Patient did not receive any neoadjuvant therapy	**0.37**	86
2B						**2.95**	129
3A	IB	Yes	-	Post-op	First line neoadjuvant FOLFIRINOX	**1**	222
3B						**8.9**	119
4A	IB	No	-	Post-op	First line neoadjuvant FOLFIRINOX, followed by capecitabine plus XRT 30Gy/10 fxs	**6.9**	153
4B						**9.2**	108
5A	IIA	No	Liver	Post-op	First line neoadjuvant FOLFIRINOX, followed by capecitabine plus XRT 30Gy/10 fxs	**6.3**	103
5B						**8.2**	104
6A	IB	Yes	Liver	Post-op		**4.58**	123

6B					First line neoadjuvant FOLFIRINOX, followed by 5 FU XRT 50.4Gy/28 fxs	**4.5**	108
7A	IIB	Yes	-	Pre-op	First line neoadjuvant FOLFIRINOX, switched to Gemcitabine abraxane for followed by capecitabine plus XRT 30Gy/10 fxs	**7.3**	94
7B						**24.9**	106
8A	IIB	Yes	Liver	Post-op, post recurrence	First line neoadjuvant FOLFIRINOX, followed by capecitabine plus XRT 30Gy/10 fxs	**5**	223
8B						**4.12**	155
9A	III	Yes	-	Post-op	First line neoadjuvant FOLFIRINOX, switched to Gemcitabine abraxane for followed by capecitabine plus XRT 30Gy/10 fxs	**0.25**	**NA**
9B						**2.6**	**NA**
10A	IA	No	-	Post-op	First line neoadjuvant FOLFIRINOX, followed by SBRT 36Gy/5 fxs	**9.2**	126
10B						**82.1**	161

A. Before therapy, B. After therapy. NA, not available data * **using AJCC 8 for the resected specimen**

Supplementary Figure Legends

Supplementary Figure S1. Graphical Abstract: Immunosuppressive role of HA-PDAC sEVs in the tumor microenvironment. The gradient scale indicates the normal PDAC to lower HA concentration, some cytokines are highlighted* due to their susceptibility to the HA.

Supplementary Figure S2. **Chemotherapy enhances sEVs secretion by pancreatic cancer cells.** Pancreatic ductal adenocarcinoma cells were treated with chemotherapeutic drugs gemcitabine (0.15 μM) and paclitaxel (0.015 μM) for 16 hours and sEVs isolated were characterized by (A) transmission electron microscope (B) nanoparticle tracking analysis (NTA) by zeta view. (C) Quantification of sEVs by NTA. The fold increase in the number of sEVs secreted following exposure of cells to drugs are shown. Data are represented as fold change ± SD, from 3 independent experiments. * $P<0.05$ vs control cells. (untreated).

Supplementary Figure S3. **Gating analysis strategy for enriched monocytes subpopulations and marker expression determination.** (A) Representative purity ratio of monocytes enriched from PBMCs from normal donors 70%, CD14+ Lymphocyte linages negative population ~82.8%. Analysis performed by flowJo-v10.7.1. (B) Representative gating analysis strategy for different marker expression on monocyte subpopulations identified by CD14 and CD16. From the live population (LD) total CD14+CD16+ were selected, then to exclude other non-monocytic populations we include a gating of HLA-DR and CD16+ cells, finally the selection of the

subpopulations was assessed. CD64, PD-L1, CD86 and HLA-DR were analyzed by percentage and MFI, respectively.

Supplementary Figure S 4. Total PD-L1 expression remains unchanged in monocyte subpopulations but increase the inflammatory cytokine profile when exposed to sEVs. (A) Monocyte subpopulations cell percentages, enriched from PBMCs healthy donors and exposed to sEVs isolated from human PDAC cells AsPC1, HPAF-II, and PANC-1 analyzed by flow cytometry. NS: non-stimulated with sEVs. Data normalized with NS and analyzed by flow cytometry in flowJo-v10.7.1, all the values below 100 events in the parental gate were not included for subgating, graphs show ± SD from seven independent experiments, statistical significance was assessed using ANOVA test and Dunn's multiple comparison test. (B) Cytokines from 3 independent normal donors were obtained by Luminex from monocytes supernatants and cell lysates, overnight co-cultured with PDAC EVs, 2 control EVs non-cancerous cell lines (HPDE and HPNE), and without EVs (NS). Results were performed in triplicate. One-way ANOVA, post hoc Tukey's test. * $p < 0.05$, ** $p < 0.01$. Full statistical analysis was included in supplementary table 1.

Supplementary Figure S5. Monocyte behavior in the presence of hyaluronan-low PDAC sEVs.

(A) PDL-1 expression in monocyte subtypes induced by PADC-derived sEVs with low HA content. sEVs not depleted of HA served as the control. Data normalized with NS and analyzed by flow cytometry in flowJo-v10.7.1, all the values below 100 events in the parental gate were not

included for subgating, graphs show ± SD from seven independent experiments, statistical significance was assessed using ANOVA test and Dunn's multiple comparison test. (B) Correlation analysis of HLA-DR and PD-L1 in PANC1 cell-derived sEVs (control or HA-low) in classical and intermediate monocyte subtypes, simple linear regression was used for statistical analysis. (C), Cytokines from 3 independent normal donors were obtained by Luminex from monocytes supernatants and cell lysates, overnight co-cultured with PDAC EVs with (red) or HA-low (blue), full statistical analysis included in supplementary table 1.

Supplementary Figure S6. **Expression of genes involved in hexosamine biosynthetic pathway and hyaluronan synthesis in PDAC patients.** mRNA expression of GFPT1, GFPT2, HAS2 and HAS3 genes in PDAC patients (n=179) compared to healthy controls (n=171). Data extracted from GEPIA database.
